# Microfibril Orientation Dominates the Microelastic Properties of Human Bone Tissue at the Lamellar Length Scale

**DOI:** 10.1371/journal.pone.0058043

**Published:** 2013-03-05

**Authors:** Mathilde Granke, Aurélien Gourrier, Fabienne Rupin, Kay Raum, Françoise Peyrin, Manfred Burghammer, Amena Saïed, Pascal Laugier

**Affiliations:** 1 UMPC Univ Paris 6, UMR 7623, Laboratoire d’Imagerie Paramétrique, Paris, France; 2 CNRS, UMR 7623, Laboratoire d’Imagerie Paramétrique, Paris, France; 3 Laboratoire Interdisciplinaire de Physique, UMR 5588 CNRS, Université Joseph Fourier Grenoble, Saint Martin d’Hères, France; 4 European Synchrotron Radiation Facility, Grenoble, France; 5 Julius Wolff Institute & Berlin-Brandenburg School for Regenerative Therapies, Charité-Universitätsmedizin Berlin, Berlin, Germany; 6 CREATIS INSERM U1044; CNRS 5220; INSA Lyon; Université de Lyon, Villeurbanne, France; University of Notre Dame, United States of America

## Abstract

The elastic properties of bone tissue determine the biomechanical behavior of bone at the organ level. It is now widely accepted that the nanoscale structure of bone plays an important role to determine the elastic properties at the tissue level. Hence, in addition to the mineral density, the structure and organization of the mineral nanoparticles and of the collagen microfibrils appear as potential key factors governing the elasticity. Many studies exist on the role of the organization of collagen microfibril and mineral nanocrystals in strongly remodeled bone. However, there is no direct experimental proof to support the theoretical calculations. Here, we provide such evidence through a novel approach combining several high resolution imaging techniques: scanning acoustic microscopy, quantitative scanning small-Angle X-ray scattering imaging and synchrotron radiation computed microtomography. We find that the periodic modulations of elasticity across osteonal bone are essentially determined by the orientation of the mineral nanoparticles and to a lesser extent only by the particle size and density. Based on the strong correlation between the orientation of the mineral nanoparticles and the collagen molecules, we conclude that the microfibril orientation is the main determinant of the observed undulations of microelastic properties in regions of constant mineralization in osteonal lamellar bone. This multimodal approach could be applied to a much broader range of fibrous biological materials for the purpose of biomimetic technologies.

## Introduction

As many biological systems, bones are designed to optimize their mechanical properties through a multiscale hierarchical organization. Hence the macroscopic behavior at the organ level is determined by multiple factors including bone mass, architecture as well as the ultrastructure of the tissue. The latter is considered to be a major determinant of the mechanical properties [1], [2]. However, the precise nature of the structural factors determining the micromechanical properties of the tissue remains unclear. This mainly stems from the degree of hierarchy which has to be considered.

At the microscopic scale, the intense remodeling activity results in a complex microstructure. In mature human cortical bone, the tissue takes the appearance of a mosaic of osteons, or Haversian system. Each of these fundamental remodeling units is typically 100 to 300 µm in diameter [3] and consists of several concentric lamellae, approximately 5–8 µm thick, surrounding a central canal. The term ‘bone lamella’ has been defined in several different ways in the existing literature: a lamella unit can be seen as a combination of dark/bright layers as seen under polarized light microscopy [4], a combination of thin/thick layers as observed from optical microscopy [5], several sublayers [6], [7] or the tissue between two transverse orientations of the collagen fibers [8]. Hence, a consensual definition of a lamellar unit would be the basic motif of a periodic structure within the osteon. It is understood that several lamellae structures can coexist in human bone [8]. They are generally described as a complex multilayer arrangement of collagen microfibrils in the range of 50–100 nm in diameter, where the fibers are parallel within a thin sublayer and tilted with respect to the osteon axis at an angle which varies across the lamellae [4], [9]–[12]. This organization is further complicated by the presence of plate-shaped calcium phosphate (carbonated hydroxyapatite) particles of nanometer dimensions, both within and between the microfibrils [9], [13]. The elongated mineral crystals have been shown to align with their crystallographic c-axes principally oriented parallel to the long axis of the collagen fibrils [4], [9], [14]–[16] providing stiffness and strength to the more compliant and weaker collagen matrix.

Recently, the elastic properties of lamellae of human cortical bone have been investigated using scanning nanoindentation [5], [17], [18] and scanning acoustic microscopy (SAM) [11], [19]. Both nanoindentation modulus and acoustic impedance showed a periodic lamellar pattern undulating between low and high values along the radial direction in cross sections of osteons. Previous reports suggest that the observed lamellar modulation of microelastic properties is either related to a variation of mineral content measured by quantitative backscattered electron imaging (qBEI) [18], [20], [21] or to changes in lamellar sublayers orientation inferred from Raman spectroscopy [11], synchrotron radiation phase nanotomography [7] and serial backscattered electron microscopy measurements [21]. These reports suggest that both anisotropy and variation in composition could explain the lamellar level modulation of the elastic properties [22]. However, so far, these parameters have not been compared with experimental microelastic measurements. Furthermore, scalar quantities, e.g. mineral density are not sufficient to take into account the finer structural details of the tissue, e.g. its anisotropy. The influence of the dimensions and organization of the mineral platelets on the elasticity also needs to be investigated in more details, which has not been done so far.

A thorough understanding of the lamellar mechanics of the osteon requires a multiphysics investigation where the amount of mineral, the specific size and arrangement of the mineral nanoparticles, as well as the microfibrils orientation and their anisotropic elastic properties appear as key parameters. This strongly relies on our ability to achieve a quantitative characterization of the mechanical properties and of the nanostructure with a comparable spatial resolution over large regions of the same sample. Over the last two decades, a panel of techniques have been used to characterize bone structure, composition, and mechanical properties, e.g. confocal microscopy [4], polarized light microscopy [23], backscatter and transmission electron microscopy [8], [21], qBEI [24], contact microradiography [25], synchrotron radiation micro and nano computed tomography with attenuation and phase contrast [7], [26], small-angle and wide-angle X-ray scattering [27], [28], wide-angle X-ray diffraction [12], and time of flight secondary ion mass spectrometry [29]. However, there are very few experimental data acquired with a sub-lamellar level resolution [7], [11], [12], [17], [18], [21], [28], [30]. Moreover, as far as we know, no study has yet been reported directly combining measurements of the mineral orientation, the anisotropic elastic properties and the mineral content on the same sample at this length scale.

Using a novel approach, we demonstrate on human bone tissue that the microelastic properties vary periodically across lamellae and that these variations are correlated with the orientations of the mineral crystals, which in turn are assumed to indirectly reflect collagen microfibril orientation at the lamellar length scale. Our approach combines SAM, quantitative scanning small-angle X-ray scattering imaging (qsSAXSI) and synchrotron radiation computed microtomography (SR-µCT) where microelastic measurements by SAM are advantageously coupled with a local complete characterization of the content, thickness, and alignment of mineral crystals at the same locations within the same specimen, and this with a sublamellar resolution.

## Methods

### Sample Preparation

**Figure 1:**
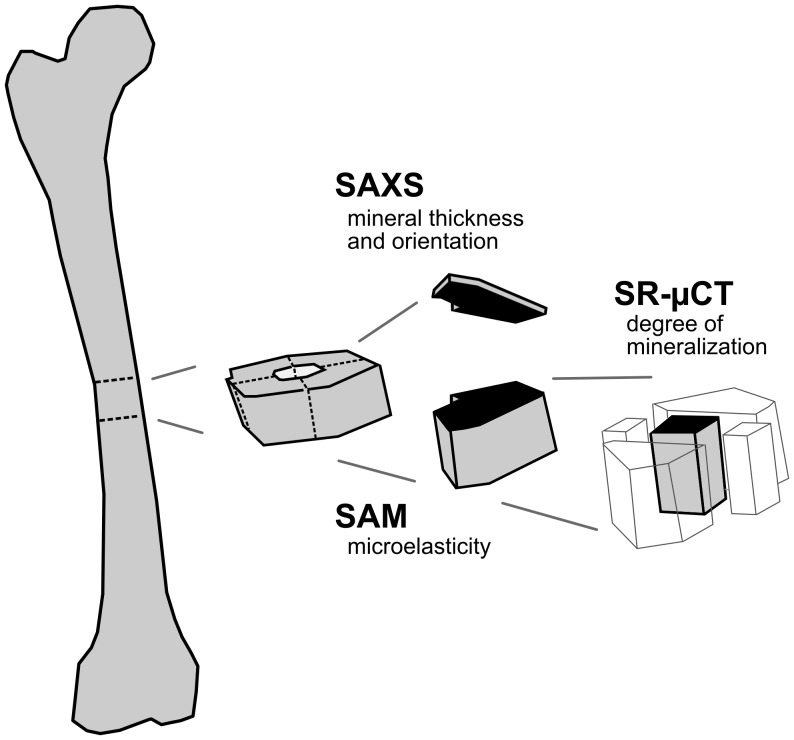
Sample preparation: the same surface is imaged with three high resolution techniques. A thin slice of 18 microns in thickness was cut at the surface of the sample block by microtomy to perform SAXS. A stick of 2×2 mm^2^ which included the region scanned by SAM was cut from the block and imaged using SR-µCT.

One important aspect of our experimental work was to consider the potential alteration of the sample by the measuring probe for each technique. While SAM is believed to be totally non-destructive, there is now a growing consensus over the potential for radiation damage of synchrotron X-ray techniques for bone and other biological materials [31]. The measuring sequence schematically described in Fig. 1 was designed to overcome this limitation while retaining the structural integrity of bone tissue, which is mandatory to correlate the results obtained from the different modalities.

The anterior quadrant of a cross-section (10 mm in thickness) was obtained from the femoral midshaft of a 92-year-old female cadaver. Ethical approval for collection of samples was granted by the Human Ethics Committee of the Centre du don des corps at the University Paris Descartes (Paris, France). The tissue donors or their legal guardians provided informed written consent to give their tissue for investigation, in accord with legal clauses stated in the French Code of Public Health.

**Figure 2:**
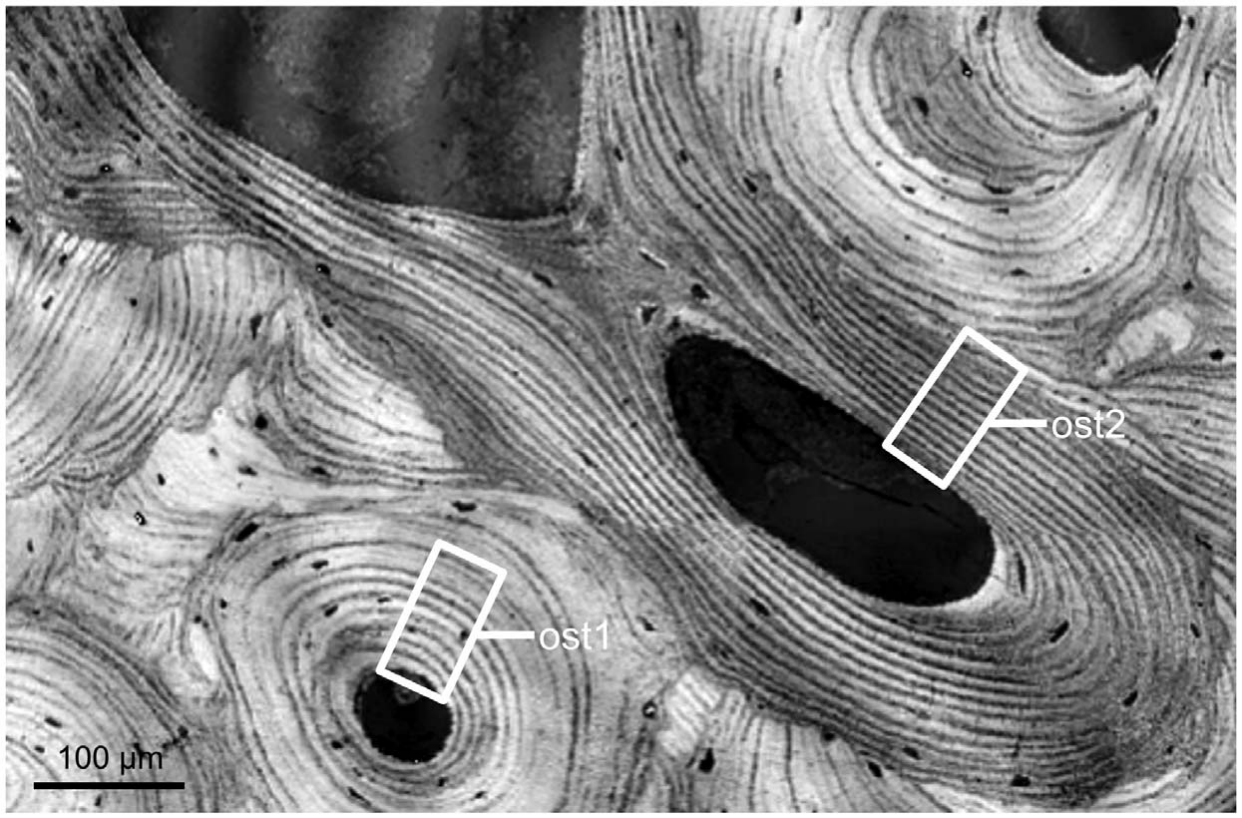
SAM image (amplitude of the reflected ultrasound beam coded in gray levels). The data analysis was performed on an identical site-matched area on each one of the two investigated osteons (namely *ost1* and *ost2*).

The sample was chemically fixed for ten days in 70% ethanol, then dehydrated in 100% ethanol and embedded in polymethylmethacrylate. A thin slice of 18 microns in thickness was cut at the surface of the sample block by microtomy (Polycut®E, Leica, Wetzlar, Germany) to perform the SAXS experiment. Two osteons within the section were carefully chosen distant from the tissue microcracks. These osteons, recognized on the surface of the remaining sample block, were then imaged with a 900-MHz scanning acoustic microscope. The surface flatness necessary for the SAM measurements was achieved by polishing the specimen sample using 1 µm diamond particle abrasive (polishing system: Logitech WG2, hard synthetic cloth: MD-Dur, Struers, Willich, Germany). Care was taken during this process to remove a minimum amount of material in order to retain the spatial correlation with the surface inspected by SAXS. Finally, a stick of 2×2 mm^2^ in cross section which included the region scanned by SAM was cut from the block using a high precision low-speed circular saw (Acutome 5, Struers Tech A/S, Copenhaguen, Denmark) and was imaged using SR-µCT. In this way, we obtained a series of SAM, qsSAXSI, and SR-µCT measurements on two osteons taken from a human femoral midshaft, namely *ost1* and *ost2* (Fig. 2).

### 900 MHz Scanning Acoustic Microscopy

Scanning acoustic microscopy (SAM) is a non-contact method that uses high-frequency ultrasound to measure the elastic response of materials. The principle has been widely described in previous work reported by our group [11], [32]. The present study used a KSI SAM 2000 (Krämer Scientific Instruments, Herborn, Germany) and a broadband lens (0.8–1.3 GHz) with a semi-aperture angle of 50°. The lens was excited with a 902-MHz burst (20 ns duration). The sample was completely immersed in distilled, degassed water temperature-controlled at 25°C. Under these conditions, the lateral resolution is approximately 1 µm and the −3 dB depth of focus is 7 µm. C-scan images were formed by displacing the transducer over the specimen surface with a 0.44 µm step increment. C-scans were acquired at decreasing probe-to-surface distances. The confocal reflection amplitude and the local surface roughness were reconstructed from the 3D data set of acoustic images using the Multi-Layer Analysis program [19]. Areas with a significant local inclination revealed by the roughness map were removed from the analysis. The microscope was calibrated with a set of six homogeneous reference materials (PMMA, polycarbonate, polystyrene, aluminum, titanium and suprasil). The reflected amplitude from the surface of the inspected bone sample was then converted into an acoustic impedance (*Z*) value, which is defined as the square root of the product of the local mass density *ρ* and local apparent stiffness coefficient *c(θ)* in the test direction *θ* [33]:
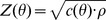
(1)


The mineralized collagen fibril is considered to be transverse isotropic with the *x_1_x_2_* plane being the plane of symmetry. The stiffness tensor *C* is:
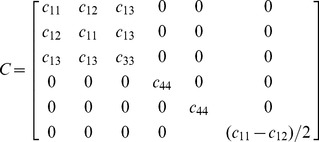
(2)where *c_11_* and *c_33_* are the elastic coefficients in the transverse and longitudinal fibril directions, respectively. The apparent stiffness at an arbitrary angle *θ* relative to the fibril long axis is:



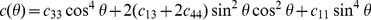
(3)
Equations (1)–(3) indicate that the acoustic impedance is determined by mass density and apparent stiffness. In the special case, when the mineral density remains constant, relative variations of the acoustic impedance directly reflect the variations in apparent stiffness. Moreover, site-matched analyses of *Z* and tissue degree of mineralization (*DMB*) obtained by SR-µCT [34], [35] have shown that the apparent stiffness of bone is strongly correlated with the acoustic impedance (R^2^ = 0.996), suggesting that acoustic impedance and stiffness are affected similarly by variations of mineral density. Therefore, the relation between *Z(θ)* and *c(θ)* can be approximated by a single regression function [36]:
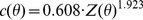
(4)


### Synchrotron Radiation Computed Microtomography

SR-µCT measurements were performed at the imaging beamline (ID19) of the European Synchrotron Radiation Facility (ESRF) in Grenoble, France. The beam energy was set at 28 keV (*λ* = 0.44 Å) by using a Si(111) double crystal monochromator. A full tomographic set of 2D images was recorded using a CCD detector (FReLoN camera; ESRF Detector group) by rotating the sample in 2000 steps within a 180° range of rotation in about 25 minutes. A comparable resolution to that of SAM and SAXS was achieved by selecting a pixel size on the detector of 1.4 µm corresponding to a 2.8 µm spatial resolution. The 3D tomographic reconstruction and the conversion of the linear attenuation coefficients values to degree of mineralization of bone (*DMB*) values were achieved as previously described [34], [37]. Finally, the slice corresponding to the surface imaged with SAM was extracted from the 3D reconstruction of the *DMB* as reported in [34].

### Small-angle X-ray Scattering

SAXS experiments were carried out on the microfocus beamline (ID13) of the ESRF. The X-ray beam was monochromatized to a wavelength *λ* = 0.997 Å (*E* = 12.437 keV) using a Si(111) double-crystal monochromator and focused to approximately 1×1 µm^2^ using a set of Kirkpatrick-Baez (KB) mirrors. A 16-bit CCD detector (MARCCD, Mar Inc., USA) with a 130 mm diameter X-ray converter screen (2048×2048 pixels of 64.45×64.45 µm^2^) was used to collect the 2D SAXS patterns. The beam center, detector tilt and sample-to-detector distance were calibrated using a silver behenate standard [38]. Regions of interest of the samples were selected using an on-axis optical microscope and scanned with a 1 µm spatial increment in horizontal and vertical directions. Thus, the scan regions were mapped with the best achievable resolution of 1 µm in direct space. The SAXS data were corrected for parasitic scattering by taking into account the transmission measured by a photodiode using the same scan parameters.

A fairly general limitation in the analysis of the SAXS data is the requirement of a partial a priori knowledge of the structure to be reconstructed to solve the missing phase information. However, in biphasic materials with sharp interfaces, several generic parameters can be calculated from the SAXS patterns without any additional structural assumptions. These so-called integral parameters relate to the total interface between the two phases *σ*, their volume fractions *φ* and the electron density contrast between the two phases *Δρ*. Two main parameters of interest were derived from the 2D SAXS patterns using a dedicated software library developed by Gourrier et al [39].

The first generic parameter that can be calculated from the scattering pattern is the average chord length *T* [40], [41]. The latter is a correlation length which, in the case of bone, can be expressed as *T* = *4φ*_M_ *φ*_O_/*σ*, where *φ*_M_ and *φ*_O_ = 1−*φ*_M_ are the mineral and organic volume fractions, respectively. If the mineral particles have dimensions a, b and c for thickness, width and height, *T* becomes *T = 2(1*− *φ_M_)/(1/a+1/b+1/c).* Many electron microscopy studies indicate that the particles are generally found in the form of thin platelets, i.e. *a<<b, c* and that thickness values fall in a relatively narrow range in human mature bone (*T* ∼ 2–4 nm) [42]–[44] as well as in a wide range of organs and species such as tooth dentin and reindeer antler [45]. The previous expression can thus be simplified to *T = 2(1*− *φ_M_)a* [46]. Hence, in zones where *φ*_M_ is constant, variations of *T* directly reflect changes in the average mineral thickness. It is noteworthy that in the transverse and longitudinal directions the dispersion in the dimensions of the platelets seems to be much more important, most likely because of the constrained mineralization inside the collagen fibrils [47], [48]. As a consequence, the particles thickness is of key importance for the characterization of the mineral phase in bone studies. Systematic differences have indeed been found in various pathologies [44], [49], [50] but the influences of the particle density, thickness and organization on the mechanical properties at the lamellar level are still poorly understood.

The second parameter is the integrated SAXS intensity *I_SAXS_*, defined as *I_SAXS_* = 2π^2^ *φ_M_φ_O_Δρ* ^2^ which can be calculated as *I_SAXS_* = ∫∫*I*(*q*,*χ*)*q* ^2^d*q*d*χ*. This quantity is generally referred to as the invariant (*Q*) in the literature since it is independent on the shape and organization of the mineral and collagen phases. In regions of constant *DMB*, *I_SAXS_* should therefore be constant to a good approximation. However, due to the strong anisotropy in the mineral nanoparticles shape, it was recently shown that, in bone, this parameter is also dependent on the orientation of the nanoparticles. This is essentially due to the fact that the integration is carried out using a single SAXS image on the detector which corresponds to a slice of the 3D reciprocal object [39].

**Figure 3:**
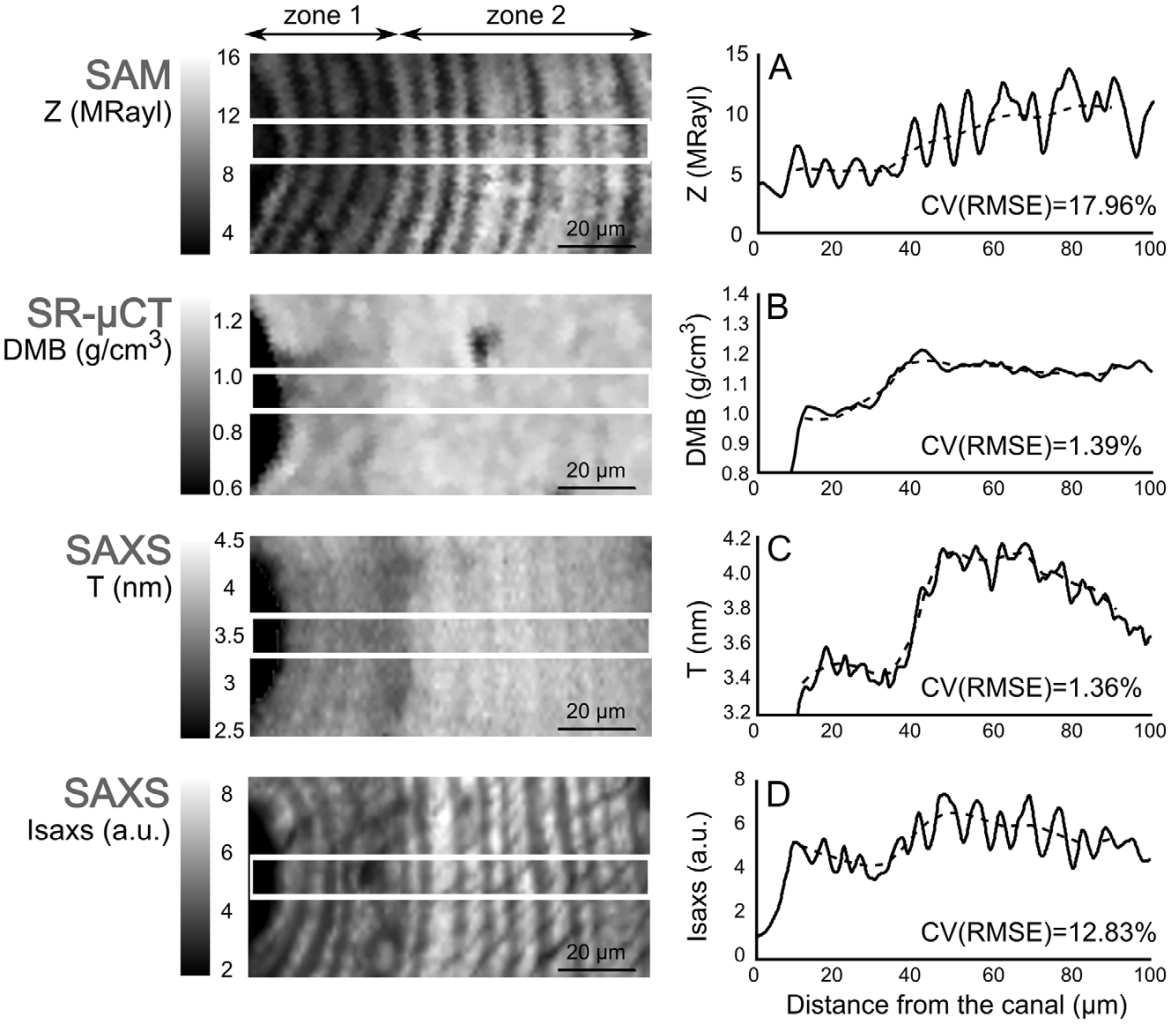
Comparison between local elastic properties and mineral ultrastructural characteristics. From top to bottom: **A** acoustic impedance (*Z*), **B** degree of mineralization of bone (*DMB*), **C** *T*-parameter linked to the mineral thickness, **D** *I_SAXS_* related to changes in the lamellar orientation (the results are illustrated for *ost1*). For each investigated parameter, the solid line represents its variations along a radial profile going from the Haversian canal to the outer lamellae. The dashed line represents the corresponding smoothed profile after averaging the values between adjacent peaks. The coefficient of variation of the root-mean-square error CV(RMSE) provides a measure of the relative amplitude fluctuations of the measured values around the averaged profile curves.

Taken alone, the information *I_SAXS_* only give access to relative variations of the average orientation of the mineral particles but does not permit a precise quantification of the angular spread. Recently, Liu et al. [27] and Seidel et al. [28] proposed a method to reconstruct the variation of the three-dimensional habit of mineral platelets within bone 3D SAXS, which combines 2D SAXS patterns collected at different angles of sample tilting. In order to establish a correspondence between the values of *I_SAXS_* obtained and the collagen microfibril angle, a similar, but simplified approach to this described by Liu et al. [27] was adopted. For this purpose, an additional scanning measurement was performed in the region shown in Fig. 3D where a line running from the central canal to the outer osteon boundary, i.e. in the radial direction, was scanned in steps of 1 µm with a beam of 500×500 nm^2^. The measurement was repeated while tilting the sample along this line in steps of 5° from –60° to +60°. It is recalled here that the maximum of *I_SAXS_* is expected when the X-ray beam is parallel to the c-axis (i.e. principal axis) of the platelets.

### Image Analysis

The measurements from the three techniques were acquired with three different pixel sizes (0.44 µm for SAM, 1 µm for SAXS, and 1.4 µm for SR-µCT). In order to facilitate the comparison between the acoustic impedance and the mineral characteristics, the four images were resampled to provide a comparable spatial resolution. Precisely, the SR-µCT and SAM images were upsampled and downsampled to a 1 µm pixel size, respectively. For each one of the studied osteons, namely *ost1* and *ost2*, an identical region of analysis (50×100 µm^2^) was site-matched on the four distributions (*Z*, *I_SAXS_*, *T-*parameter and *DMB* maps) for comparison and statistical analysis (Fig. 2). Within the zone of study, the data were averaged over ten adjacent lines (i.e. over 10 µm) and the corresponding radial profiles were analyzed (Fig. 3). The first osteon (left in Fig. 2) revealed distinct parameter values in the inner (∼0–30 µm from the canal) and peripheral areas (Fig. 3). The mean and standard deviation values were calculated on these two zones. When a layered structure was visible (i.e. on the SAM and qsSAXSI images), a spatial frequency analysis was performed to determine the mean value of the spatial oscillations. To this end, each one of the averaged signals was windowed with a hanning window and its power spectral density was computed. The mean value of the spatial oscillations is given by the inverse of the frequency at which the spectral density reaches its maximum. The local extrema (minima and maxima) of the radial profiles were determined over the nine first lamellae for *ost1* and seven lamellae for *ost2* and used to perform regression analysis on the parameters. All statistical results were considered significant for p-values less than 0.05. The statistical analysis was performed using the MATLAB™ Statistics Toolbox (The Mathworks, Inc., USA).

## Results

While a marked lamellar modulation of acoustic impedance, *I_SAXS_*, and to a lesser extent of *T* was observed on corresponding images, no such modulation could be seen on the *DMB* image (Fig. 3). As introduced above, the first osteon exhibited clearly two distinct zones for all the parameters with lower values in the inner area (zone 1) compared to the peripheral area (zone 2).

### Acoustic Impedance (Z)

**Figure 4:**
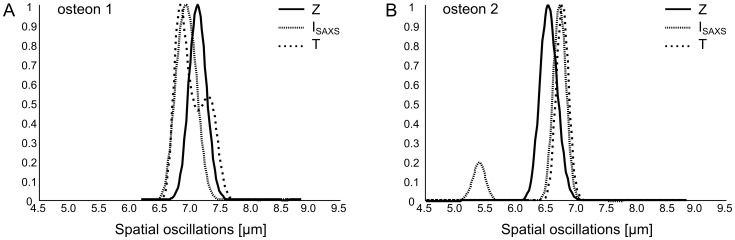
Result of the spatial frequency analysis performed on the *Z*, *I_SAXS_*, and *T* images. The two plots display the normalized power spectral density as a function of the spatial oscillations (i.e. the inverse of the spatial frequency) on *ost1* (**A**) and *ost2* (**B**).

On the high resolution SAM image, the osteonal lamellar structure was reflected in a periodic pattern which oscillated between high and low impedance values (Fig. 3A). The spatial frequency analysis revealed an average period of the SAM profile (i.e. an average thickness of the lamella) of 7.1 and 6.6 µm for *ost1* and *ost2*, respectively (Fig. 4). The local minima and maxima impedance values were 5.3±1.5 MRayl and 8.7±2.4 MRayl, respectively in *ost1*; and 4.1±0.2 MRayl and 5.8±0.2 MRayl, respectively in *ost2*. In *ost1*, the mean impedance in zone 1 was found to be lower than in zone 2 (5.2±1.0 vs. 8.4±2.0 MRayl, respectively). In spite of the intra-lamellar variation, the acoustic impedance averaged over a lamellar period remained invariant within the zones 1, 2 of *ost1* and *ost2*.

### Degree of Mineralization of Bone (DMB)

In contrast to *Z*, the *DMB* map obtained from SR-µCT (Fig. 3B) showed no lamellar level modulation of mineral content. The first osteon revealed a lower *DMB* in the inner zone around the canal (zone 1) compared to the peripheral zone (zone 2) (1.02±0.06 g/cm^3^ and 1.16±0.04 g/cm^3^ respectively). The mean value of the mineral density was 1.08 g/cm^3^ (1.10±0.08 g/cm^3^ and 1.01±0.05 g/cm^3^ in *ost1* and *ost2*, respectively).

### Mineral Thickness (T-parameter)

The spatial variations of the *T-*parameter are shown in Fig. 3C with the same field of view as that of *Z* and *DMB*. A similar trend as for the *DMB* was observed in *ost1* with a bimodal distribution in particle size. These were found to be significantly smaller in zone 1 than in zone 2 (3.42±0.12 vs. 4.03±0.11 nm). A slight modulation of *T* can be observed along the radial direction of the osteon, the minimal and maximal *T* values being 3.70±0.30 and 3.82±0.34 nm respectively in *ost1*; and 3.33±0.09 and 3.50±0.05 nm in *ost2*. The average period as given from the spatial frequency analysis was 6.9 and 6.8 µm for *ost1* and *ost2*, respectively (Fig. 4).

### Mineral Orientation (I_SAXS_)

The image of *I_SAXS_* was calculated in the same regions as for *Z* and *DMB* (Fig. 3D). The pattern observed in this image is very similar to that of *Z,* with a clear periodical modulation found along the radial direction with values alternating between two extrema (4.4±0.5 and 6.0±1.0 a.u. for the average local minima and maxima, respectively in *ost1*; and 3.4±0.2 a.u. and 4.7±0.4 a.u. in *ost2*.). The average period of the modulations as given by the spatial frequency analysis was 6.9 and 6.7 µm for *ost1* and *ost2*, respectively (Fig. 4). It was again noticed for *ost1* that the lamellae close to the Haversian canal (zone 1) exhibited lower *I_SAXS_* values than the outer lamellae (zone 2) (4.3±0.7 vs. 6.0±1.0 a.u.).

**Figure 5:**
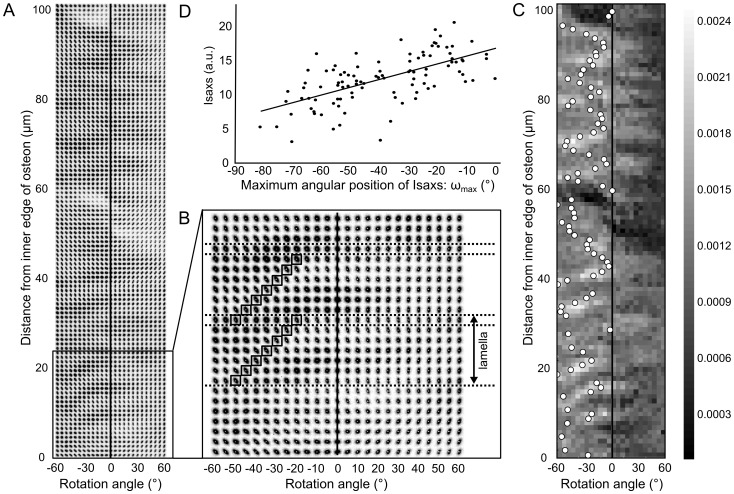
SAXS calibration of the mineral nanoparticle orientation. **A** Composite image showing the 2D SAXS pattern as a function of scan position along the radial direction of the osteon and tilt angle ω; **B** zoom in the innermost part of the scan (close to the Haversian canal); **C** map of the intensity *I_SAXS_* along the direction of the scan across the osteon as a function of the tilt angle, with the result of the fit overlaid in the form of white dots indicating a maximum. The position of the maximum was calculated by fitting the tilt profiles (from −60° to 60°) at each scan point by a sine function; **D** correlation between *I_SAXS_* and the angular position of the maximum *I_SAXS_* measured when tilting the sample between –60° and +60°.

A composite image showing the 2D SAXS patterns as a function of the rotation angle about the radial direction and position across the osteon is shown in Fig. 5A. The closer examination in the innermost part of the scan, i.e. in the part closer to the Haversian canal, shows that each horizontal row of the SAXS patterns is somewhat shifted to the right within a vertical stack of six rows. This is evidenced in Fig. 5B for two such stacks where a SAXS pattern with approximately the same shape and intensity is highlighted by a box for each row in the stack. This tends to indicate, qualitatively, a tilt of the 3D SAXS signal by ∼ 5° for each step of 1 µm along the radial direction within 6–7 µm. The data were normalized to account for changes in apparent volume probed by the micro-beam as a function of tilt angle and the values of *I_SAXS_* were calculated for each scan position (Fig. 5C). The angular profiles obtained were then fitted using a periodic function to determine *ω_max_*, the angular position of the maximum of *I_SAXS_* (overlaid white dots in Fig. 5C) which, assuming a prolate ellipsoid form for the 3D SAXS signal [28], gives the orientation of the mineral platelets (and thus of the mineralized fibers) perpendicular to the radial direction, i.e. within the lamellar plane.

Two main results were obtained from this analysis. First, the orientation map was not symmetrical: the values of *ω_max_* were found to fall within a restricted angular interval of ∼ 0–60°, where 0° represents the normal to the sample plane. Furthermore, despite the non-negligible amount of irregularity in the data (r = −0.78), the correlation of I_SAXS_ and the angular position of the maximum *ω_max_* could be seen as ‘master curve’ to calibrate the integrated SAXS intensity with the orientation of the mineral particles and thus the collagen fibrils (Fig. 5D).

### Acoustic Impedance Versus Mineral Characteristics

In contrast to the pronounced modulations of *Z*, *I_SAXS_*, and to smaller extent *T*, the *DMB* distribution across the evaluated osteons did not exhibit any lamellar pattern. In particular, it indicated that the sublamellar level variations of *Z* were not caused by *DMB* variations.

When the mean value of the parameters over one lamella was considered, the comparison between the inner and peripheral zones of the first osteon showed that an increase of *DMB* was associated to higher acoustic impedance, *I_SAXS_* and *T*-parameter values.

**Figure 6:**
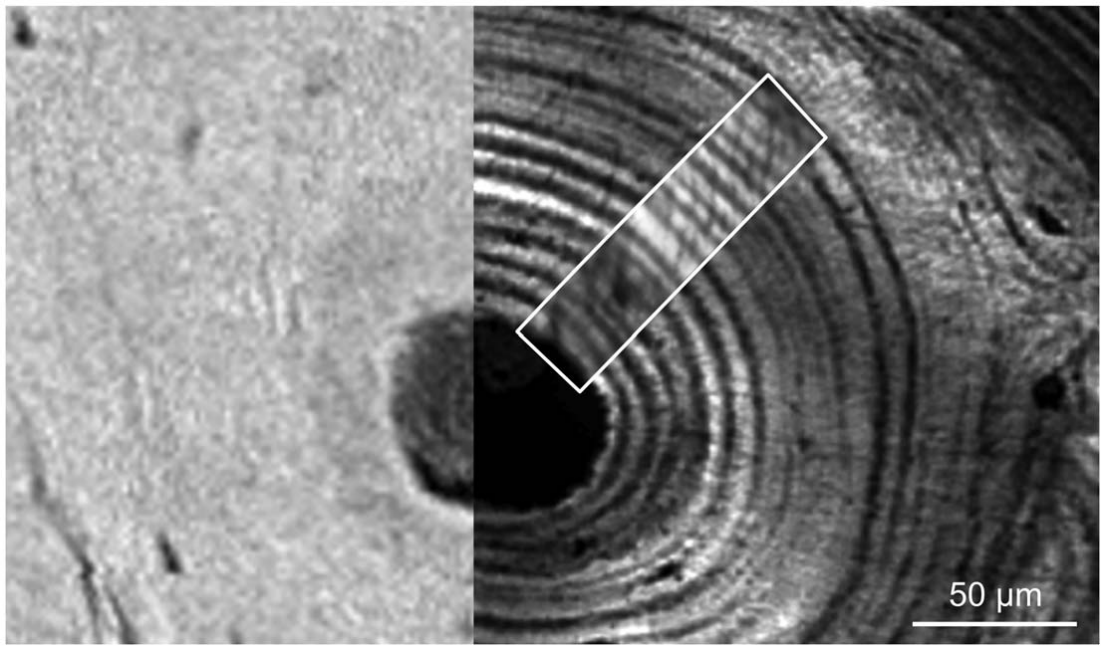
Site matched images on *ost1*: *DMB* map (left), *Z*-map (right, background) and *I_SAXS_* map (right, foreground).

Careful comparison between SAM and SAXS images showed that the lamellar patterns (and in particular the extreme values) of *Z*, *I_SAXS_* and *T* spatially coincided (see the match for *Z* and *I_SAXS_* on Fig. 6). The spatial frequency analysis performed on both the SAM and SAXS images confirmed that *Z*, *I_SAXS_* and *T* exhibit a similar spatial periodicity of approximately 7 µm, corresponding to the average thickness of one lamella (Fig. 4).

**Figure 7:**
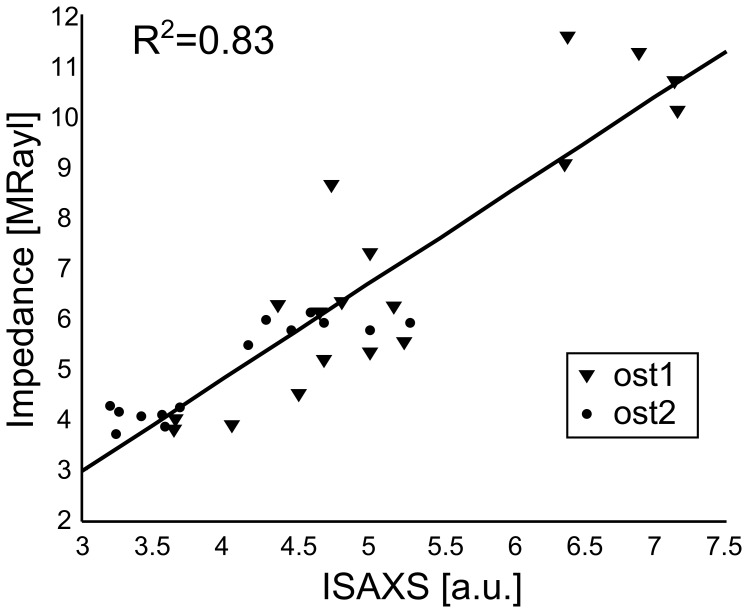
Minima and maxima of acoustic impedance (*Z*) and mineral orientation (*I_SAXS_*) show a positive correlation (linear regression for the pooled data from *ost1* (▾) and *ost2* (•).

Successive pairs of maxima and minima in the SAM and SAXS profiles on *ost1* and *ost2* were selected for a regression analysis (Fig. 7). A strong and positive correlation was found between *Z* and *I_SAXS_* (*R* ^2^ = 0.83, p<10^−5^).

## Discussion

The present work aimed to determine the impact of the ultrastructural characteristics of bone tissue on its local elastic properties as derived from acoustic microscopy measurements. To address this question, we combined for the first time the SAM, SAXS and SR-µCT techniques in order to compare, at the scale of a lamella, site-matched information on bone microelastic properties, mineral content as well as crystals thickness and orientation.

Our results reaffirm that osteons exhibit a radial modulation in acoustic impedance which alternates between high and low impedance values as reported previously, e.g. by Hofmann et al. [11]. This periodic pattern can be related to the osteonal lamellar structure. In our study, an osteonal lamella is defined by a period as imaged via SAM, which thickness of about 7 µm fits well with reported values observed with different techniques [4], [11], [12]. This period coincides with the period of the SAXS images which is an additional illustration of the fact that lamellae can be defined by the structural organization of the mineral platelets and, hence, the collagen microfibrils. However, although variations of the mechanical properties and mineral characteristics exist within a lamella, they tend to disappear when we consider the same properties averaged over the entire lamella. As an illustration, the mean acoustic impedance over a single lamella remained invariant within regions of constant *DMB* across the osteon, which is in agreement with nanoindentation results [18].

The abrupt increase in the mineralization of the first osteon between zone 1 and zone 2 led to a marked increase in acoustic impedance, which is consistent with the theoretical relationship between *Z* and the local mass density. Despite the step-like increase, the mineral density was found relatively invariant in the sample, with a mean *DMB* of 1.08 g/cm^3^, in agreement with reported values on human cortical bone [25], [37], [51]. Such a homogeneous distribution of the bone mineral content within the osteon has also been observed using Raman spectroscopy [11], [52], synchrotron radiation X-ray phase nanotomography [7], qBEI [53] and time of flight secondary ion mass spectrometry [29]. The uniform mineral density within zone 1 and zone 2 of *ost1* and within *ost2* confirm that impedance variations in apparent stiffness that cannot be explained by variations in mineral content. Here, we point out that, although the tissue mineralization is known to be correlated with tissue stiffness [18], [35], it does not necessarily explain the prominent variation in elasticity at the sub-lamellar scale, confirming our search for other factors. Moreover, it should be noted that small intra-lamellar variations of tissue mineralization, as reported by others [20], [21] can also be seen in the *DMB* maps, e.g. in the lower part of Fig. 3B, directly adjacent to the Haversian canal. Such density fluctuations also contribute to the variations of *Z*, but could generally not explain the strong intra-lamellar modulation observed in the impedance image (Fig. 6).

The SR-µCT measurements allow for a better evaluation of the relationship between the *T*-parameter and the actual average thickness of the mineral particles within the probed volume. In this regard, we recall that *φ_M_ = DMB/ρ_M_*, where *ρ_M_* is the mass density of mineral particles, such that *T = 2(1- DMB/ρ_M_)a*. Taking the *DMB* values for zones 1 and 2 as 1.02 and 1.16 g.cm^−3^ and *ρ_M_ ∼ 3 g.cm* ^−*3*^ [54], we find that Δ*a* = 0.69 nm ∼Δ*T* = 0.61 nm. Hence, in the remaining discussion, variations of *T* will be taken as a good approximation of variations of the particle thickness. A slight modulation of *T* was observed along the radial direction of both osteons. With a constant *DMB*, this small modulation could either be interpreted as a variation in particle density, or as an orientation effect. Conceptually, there is no a priori reason to believe in a variation in particle density within a single lamella, unless there be a concomitant, albeit very weak fluctuation in *DMB*. Although this cannot be fully excluded, we do not observe such change within the limits of our detection scheme, i.e. by assessing the *DMB* from the X-ray linear attenuation coefficient with a spatial resolution of 2.8 µm. The average period of the modulations in *T* was close to that of *I_SAXS_* with 6.9 and 6.8 µm for *ost1* and *ost2*, respectively, and a significant correlation was found between the two parameters after adjustment for *DMB* (R^2^ = 0.49, p = 0.002), such that an orientation effect seems more likely.

The last investigated parameter, *I_SAXS_*, provides information about the relative changes in orientation of microfibrils in the case of constant density. Our study is the first to propose a calibration of the integrated SAXS intensity in order to establish a correspondence between the obtained values of *I_SAXS_* in a full scan region and the corresponding collagen microfibril angle. From this calibration, we were able to conclude that the fibrils are oriented within a restricted angular interval (between −60° and 0° with respect the normal direction of the sample surface) which is consistent with previous findings [12], [28]. Furthermore, it can be observed that, although there is a regular trend in the organization of the nanocrystals, there is also a non-negligible dispersion in the data (Fig. 5D). This could be explained by either or both the sample preparation and the biological variation in biomineralization and microfibril deposition during the bone remodeling processes. It is believed that intrafibrillar mineral platelets are more uniformly ordered along the fiber direction than extrafibrillar mineral [22], [55]. The relative fraction of extrafibrillar minerals may be one of the factors explaining the high amount of scattering in the calibration curve. Moreover, in Fig. 3D, faint lines can be observed running in a diagonal direction to the lamellae, which could, in fact, indicate the presence of micro-cracks induced by the microtoming process, such that the sample preparation is also a probable explanation. Nevertheless, the negative correlation between *I_SAXS_* and the absolute value of *ω_max_* allow us to conclude that high values are found where the microfibrils are closer to the surface normal direction, while lower values point to an important tilt angle. Although a more precise estimation of the absolute mineral particle orientation could be obtained by the methods described by Liu et al. [27] or Wagermaier et al. [56], the procedure used in this study is considerably simpler and thus provides a quick and efficient way of evaluating changes in orientation along the normal to the radial direction in order to calibrate the images of *I_SAXS_*.

The strong and positive correlation between the acoustic impedance and the SAXS intensity confirms that, in regions of invariant mineral density, the fibril orientation is the main factor contributing to the apparent elastic variations at the sublamellar level. The influence of the fibril orientation on the elastic properties was expected from mechanical considerations. It results from the fact that mineralized fibrils are mechanically anisotropic (the fibrils are much stiffer in the axis direction than perpendicular to it [11], [15], [32]). Thus, changes in fibril orientation within a lamella would likely lead to a local variation of the elastic properties. Our research demonstrated experimentally for the first time this theoretical inference by exhibiting a high correlation between the elastic properties and the fibril orientation over the osteon. Furthermore, the *I_SAXS_* calibration confirms that the higher stiffness values, reflected by higher acoustic impedance values, are associated with a more longitudinal orientation of the fibers, i.e. with fibers oriented perpendicularly to the sample surface.

Our experimental observations merit further investigations to clarify a model for the lamellar structure. Numerous models have been proposed but no one is comprehensive and supported by a rigorous experimental validation. Reisinger et al. [57] recently examined the theoretically predicted elastic anisotropy of bone lamellae as a function of fibril orientation pattern as given by four different models from the literature: (i) the orthogonal plywood (two orthogonal sublayers oriented at +45° and −45°) [8], (ii) the twisted plywood (the fibril rotation angle changes linearly throughout the lamella from −90° to +90°) [8], (iii) the 5-sublayer pattern of Weiner et al. [6] (a single lamella is asymmetric and composed of 5 sublayers of different orientation (30°,0°,−30°,−60°,−90°) and thickness (40%,40%,7%,7%,7%) of the lamellar unit respectively), and (iv) the orientation pattern based on diffraction measurements of Wagermaier et al. [12] (the angle of the fibers orientation changes from about −10° to −60° relative to the long axis of the osteon with a periodicity of 5–7 µm). They concluded that two of the four models would be coherent with experimental results: the 5-sublayers pattern proposed by Weiner et al. [6] and the SAXS/WAXD fibril orientation pattern by Wagermaier et al. [12]. The *I_SAXS_* calibration provides data that are consistent with Wagermaier’s model [12] with the same amplitude range of fibers orientation. However, the micrometric resolution of our study prevents us from confirming a particular model since any sublayer less than a micron would likely not be resolved with our techniques (e.g. considering a 7 µm lamella in the 5-subplayer pattern, each one of the three sublayers oriented at −30°, −60° and −90° would be less than 500 nm thick). Recently introduced serial 2D BSE microscopy [21] and SR phase nanotomography [7] methods have the potential to probe larger tissue sections in 3D with unprecedented resolution and fibril orientation.

In considering the limitation of this study, we recognize the small size of explored samples. Due to technical and time limitations, it was not possible to perform correlated measurements of mechanical and mineral properties on a larger number of osteons. Given the significantly strong correlation found between impedance and mineral crystal orientation, measurements over a region containing many osteon types may not weaken our interpretations but would certainly be advantageous to explore the inter-osteonal variability (attributed to bone remodeling) and the dependence of correlated variables with bone anatomical site. In particular, polarized light studies associated with mechanical testing (compression, tension, bending, torsion) have demonstrated that each osteon exhibits specific mechanical properties related to the orientation of the collagen fibers, which appears to be well adapted for handling different types of stresses *in vivo* [58]–[61]. In this regard, future work will be designed to investigate different schemes, such as an osteon under a load shielded situation, or tissue from a very young individual, where stress directionality has not developed yet.

A further limitation is the tissue preparation protocol used in this study. Because the sample had to be measured at different time points and different locations, PMMA embedding was chosen to preserve the samples microstructural and mechanical integrity. Although our preparation protocol is commonly used, both for ultrastructral and micromechanical investigations [12], [18], [52], [62]–[64], the ethanol fixation and embedding process may have biased the acoustic impedance values. Using apatite nanocrystals with very close composition to bone mineral, it has been shown that most modifications occur on the surface hydrated layer upon dehydration, such that the particle core is preserved [65]. Furthermore, bone minerals platelets in mature bone tissue fuse together [66], so the replacement of water by the embedding resin is not believed to alter the structural arrangement of the mineralized fibrils. SAXS measurements in mature bone have confirmed that ethanol treatment preserves the fibril nanostructure, i.e. the typical 67 nm periodicity of the tertiary structure collagen I molecules [67]. However, it alters the material stiffness due to cross-linking of proteins and other molecules. The replacement of water by the embedding resin results in higher nanoindentation moduli [5], [18], and acoustic impedance values [68]. Thus, although the measured impedance values are presumably biased, our main observation of the synchronous intralamellar modulation of acoustic impedance and mineral orientation is expected to remain valid.

In summary, we demonstrate the possibility for assessing structural-functional relations in human cortical bone at the lamellar scale by combining three high resolution imaging techniques. The proposed experimental protocol allows for the concurrent assessment of mineral characteristics (particles orientation, thickness and volume fraction) which are likely to determine the local stiffness. An alternating pattern of high and low impedance values across a human osteon was found in spite of a locally homogeneous distribution of mineral quantity. Our results provide direct evidence that, for a relatively constant mineral density, the main factor contributing to the elasticity variations at the lamellar scale is the platelet orientation reflected in the modulations of the integrated SAXS intensity. Despite the specimen size limitation, our study opens doors to new investigations since variations (e.g. of composition, elastic properties or fiber orientation) are expected depending on the type of tissue, the anatomical site, the age, or the pathologic conditions.
